# Factors associated with the intention to participate in activities during a nuclear disaster situation among firefighters

**DOI:** 10.1093/jrr/rraa061

**Published:** 2020-08-07

**Authors:** Keita Iyama, Yoshinori Takano, Tsukasa Takahashi, Arifumi Hasegawa

**Affiliations:** Department of Radiation Disaster Medicine, Fukushima Medical University, Fukushima, Japan

**Keywords:** intention, nuclear disaster activity, training student, firefighter, human resources

## Abstract

Willingness to participate in activities during a nuclear disaster situation among firefighters is low. Thus, we aimed to identify the factors affecting the intention to participate in nuclear disaster activities. A questionnaire survey was conducted among firefighter training students (*n* = 186) and firefighters (*n* = 410), and a multivariate logistic regression analysis was performed to identify factors affecting the intention to participate in nuclear disaster activities. The percentage of students and firefighters who were willing to participate in nuclear disaster activities was 70.4% (*n* = 131) and 56.3% (*n* = 231) (*P* < 0.01), respectively. The factors affecting the students’ intention to participant were “wish to learn more information about radiation” and “firefighters should actively work in a nuclear disaster.” Meanwhile, the factors affecting the firefighters’ intention to work were “have self-confidence during nuclear disaster activities,” “participate if there is an incentive,” “unable to get a family member to understand the need to participate in a nuclear disaster activity,” and marital status. A student’s decision might be strongly connected to social norms about participating in nuclear disaster activities. The willingness to participate in nuclear disaster activities among firefighters might be improved by facilitating activities that can build their self-confidence, providing sufficient incentives, and helping their families understand their work. Therefore, not only direct education for responders but also educational activities for the general public and their families are essential.

## INTRODUCTION

The general public tends to believe that healthcare providers, firefighters, and other professionals with similar jobs who participate in disaster activities are willing to work as crisis responders in any type of disaster. On the contrary, they are not always willing to work in every disaster situation, and their willingness or motivation to engage in a disaster activity depends on the type of disaster [1, 2]. Lanzilotti *et al*. emphasized that ~90% of healthcare workers are willing to work during a general disaster and only 40% during a nuclear disaster situation [1]. Similar results were observed among firefighter training students (students), indicating that students are willing to work during disasters [3]. In this study, most students wanted to work during general disasters (94.5%, building or house fire; 90.6%, earthquake) after graduation. However, only 42.4% wanted to work during disasters involving nuclear accidents. Furthermore, a recent study revealed that dentists were motivated to provide assistance during a disaster but did not take actual action or show preparedness [4]. Another study revealed that paramedics were more anxious about nuclear radiation events than other events [5].

This type of mentality may lead to a decrease in human resources responding to a disaster. During a nuclear disaster, human resources are always limited. In addition, a low level of motivation among responders may affect the smooth functioning of the disaster response system. Therefore, not only the number of people willing to work during a nuclear disaster but also their motivation to deal with the abovementioned disasters must be increased. To address these problems, the factors affecting the willingness to work during a nuclear disaster must be identified.

In order to determine methods of increasing the number of human resources and improving the intention to engage in a nuclear disaster, we aimed to (i) understand the mindset of a person with a strong intention to engage in nuclear disaster activities and (ii) identify the measures required to increase participation. We believe that proposing countermeasures for both positive and negative factors associated with the intention to participate in nuclear disasters will help to create an environment in which crisis responders can more readily engage. Thus, in this study, we conducted a questionnaire survey among firefighters, representing a crisis responder, and students, who would be firefighters in the future. We aimed to identify the factors associated with the intention to actively participate in nuclear disaster activities and to propose measures to improve this intention. Moreover, examples of educational activities that can improve participation of human resources in a nuclear disaster activity are presented.

## MATERIALS AND METHODS

An anonymous questionnaire survey was conducted from July 2019 to September 2019. The questionnaire sheet was distributed to all students (*n* = 211) from two firefighter training schools in a nuclear disaster-affected area and a non-affected area with a power plant, as well as to all employees (*n* = 516) of two fire departments in a nuclear disaster-affected area. The effective response rate of students and firefighters was 88.2 and 79.5%, respectively, and 186 students and 410 firefighters were finally included in the analysis. Significant differences were observed between the students and firefighters in terms of age [19 (19–20) vs 34 (28–45) years, respectively, *P* < 0.001], female population [28 (15.1%) vs 4 (1.0%), respectively, *P* < 0.001] and marital status [1 (0.5%) vs 305 (74.4%), respectively, *P* < 0.001] (Table 1).

**Table 1:** Characteristics of study participants

	Total (*n* = 596)	Students (*n* = 186)	Firefighters (*n* = 410)
Age, median, years (IQR^a^)	29 (20–39)	19 (19–20)	34 (28–45)
Sex, female *n*,(%)	32 (5.4)	28 (15.1)	4 (1.0)
Marital status *n*,(%)	306 (51.3)	1 (0.5)	305 (74.4)

^a^IQR, interquartile range.

We created 23 questions and used a four-point Likert scale (strongly agree, agree, disagree, and strongly disagree). In the subsequent analysis, the following answers were used: yes, if a participant strongly agrees or agrees and no, if a participant strongly disagrees or disagrees. The questionnaire is presented in Table 2. To clarify the intention to participate in nuclear disaster activities, the answer to the following question was considered the outcome: “Q1: I want to actively participate in response activities during a nuclear disaster situation.” First, a univariate logistic regression analysis was performed to assess the significance of Q1 and the other questions (Q2–Q23, age and marital status). Based on the results, questions with significant differences and age were included in the subsequent multivariate logistic regression analysis to identify the factors affecting the intention to participate in nuclear disaster activities. We also used a variance inflation factor (VIF) to check for multicollinearity. All statistical analyses were performed using JMP 14 (SAS Institute Inc., Cary, NC, USA), and the significance level was set at 0.05.

**Table 2:** Results of the questionnaire survey

		Total (596) Yes, *n* (%)	Students (186) Yes, *n* (%)	Firefighters (410) Yes, *n* (%)	*P*-value
Q1	I want to actively participate in response activities during a nuclear disaster situation.	362 (60.7)	131 (70.4)	231 (56.3)	0.001
Q2	I want to actively receive education or training in nuclear disasters.	479 (80.4)	138 (74.2)	341 (83.2)	0.011
Q3	I want to participate in seminars on nuclear disasters.	442 (74.2)	117 (62.9)	325 (79.3)	<0.001
Q4	I want to actively work on nuclear disaster countermeasures.	432 (72.5)	130 (69.9)	302 (73.7)	0.340
Q5	I want to learn more about radiation to prepare for a nuclear disaster	475 (79.7)	142 (76.3)	333 (81.2)	0.170
Q6	Preparing for nuclear disasters (e.g. education or training) is more important than preparing for other general disasters.	466 (78.2)	156 (83.9)	310 (75.6)	0.024
Q7	Preparing for nuclear disasters (e.g. education or training) has been prioritized more than preparing for other general disasters.	341 (57.2)	131 (70.4)	210 (51.2)	<0.001
Q8	Nuclear disasters can occur in the area where you work or live.	380 (63.8)	67 (36.0)	313 (76.3)	<0.001
Q9	I have self-confidence in nuclear disaster activities.	134 (22.5)	22 (11.8)	112 (27.3)	<0.001
Q10	I am anxious about firefighter activities in a nuclear disaster situation.	492 (82.6)	154 (82.8)	338 (82.4)	0.915
Q11	It is helpful to prepare for a nuclear disaster by receiving education or training.	575 (96.5)	179 (96.2)	396 (96.6)	0.831
Q12	I will participate in nuclear disaster activities if there are incentives, such as insurance and special salaries.	400 (67.1)	154 (82.8)	246 (60.0)	<0.001
Q13	Education and training are indispensable for nuclear disaster activities.	552 (92.6)	165 (88.7)	387 (94.4)	0.014
Q14	Preparing for a complex disaster, mainly a nuclear disaster, is essential in protecting citizens.	574 (96.3)	179 (96.2)	395 (96.3)	0.950
Q15	Firefighters should actively work in a nuclear disaster situation.	528 (88.6)	170 (91.4)	358 (87.3)	0.147
Q16	If my colleagues are preparing for nuclear disasters (e.g. education or training), I should take action as well.	490 (82.2)	152 (81.7)	338 (82.4)	0.832
Q17	Firefighters should be routinely educated and trained to meet the expectations of citizens.	567 (95.1)	178 (95.7)	389 (94.9)	0.666
Q18	If firefighters actively prepare for nuclear disasters, citizens will have a good impression of the firefighters’ actions.	439 (73.7)	151 (81.2)	288 (70.2)	0.005
Q19	At your own workplace or school, it is easy to obtain information about seminars for nuclear disaster response.	350 (58.7)	59 (31.7)	291 (71.0)	<0.001
Q20	My workplace or school holds seminars for nuclear disaster response.	241 (40.4)	55 (29.6)	186 (45.4)	<0.001
Q21	In my environment I can learn about radiation.	343 (57.6)	81 (43.6)	262 (63.9)	<0.001
Q22	It is impossible to get a family member to understand the need to work in a nuclear disaster situation.	218 (36.6)	49 (26.3)	169 (41.2)	<0.001
Q23	I feel sorry for my family if I get exposed to radiation.	456 (76.5)	149 (80.1)	307 (74.9)	0.163
	Marital status	306 (59.6)	1 (0.5)	305 (74.4)	<0.001

## RESULTS

The percentage of students and firefighters who were willing to participate in nuclear disaster activities and who answered yes to Q1 was 70.4% (*n* = 131) and 56.3% (*n* = 231) (*P* < 0.01), respectively (Table 2). Regarding the other questions, a high number of students answered yes and the answers for Q6, Q7, Q12 and Q18 were significant. Moreover, a higher number of firefighters answered yes and the answers for Q2, Q3, Q8, Q9, Q13, Q19, Q20, Q21 and Q22 were significant.

The univariate logistic regression analysis showed that Q2–5, Q12 and Q15–17 were considered significant factors for the students’ intention to participate in nuclear disaster activities. In addition, Q2–9, Q11–22 and marital status were found to affect the firefighters’ intention to participate in such activities (Table 3). These factors were included in the subsequent multivariate logistic regression analysis. Then, 8 and 21 factors associated with the students’ and firefighters’ intention to participate, respectively, were included as independent variables. Every derived VIF value of the model was <3 (maximum VIF value: 2.504); in addition, we considered that there was no multicollinearity in this analysis model. Based on multivariate logistic regression analysis, the factors affecting the students’ intention to work in a nuclear disaster situation were as follows (Table 3): “Q4: I want to actively work on nuclear disaster countermeasures” [odds ratio (OR): 2.9, 95% confidence interval (CI): 1.1–7.8, *P* = 0.034] and “Q15: Firefighters should actively work in a nuclear disaster situation” (OR: 4.9, 95% CI: 1.2–20.0, *P* = 0.026). Meanwhile, those affecting the firefighters’ intention to participate were “Q9: I have self-confidence in nuclear disaster activities” (OR: 3.2, 95% CI: 1.8–5.8, *P* < 0.001), “Q12: I will participate in nuclear disaster activities if there are incentives such as insurance and special salaries” (OR: 3.2, 95% CI: 2.0–5.3, *P* < 0.001), “Q22: It is impossible to get a family member to understand the need to work in a nuclear disaster situation” (OR: 0.6, 95% CI: 0.3–0.9, *P* = 0.019), and marital status (OR: 0.4, 95% CI: 0.2–0.7, *P* = 0.003).

**Table 3:** Results of multivariate logistic regression analysis

	Total (*n* = 596)		Students (*n* = 186)		Firefighters (*n* = 410)	
	Univariate	Multivariate	Univariate	Multivariate	Univariate	Multivariate
	OR (95%CI)	OR (95%CI)	OR (95% CI)	OR (95%CI)	OR (95%CI)	OR (95%CI)
Q2	4.1 (2.7–6.4)	1.4 (0.7–2.7)	7.5 (3.6–15.6)	1.8 (0.7–4.9)	3.7 (2.1–6.4)	1.0 (0.4–3.0)
Q3	3.9 (2.7–5.8)	1.8 (1.0–3.5)	7.2 (3.6–14.5)	2.1 (0.8–5.4)	4.2 (2.5–7.0)	2.3 (0.8–6.3)
Q4	4.5 (3.1–6.6)	***1.8 (1.0–3.3)*** ^**a**^	8.0 (3.9–16.3)	***2.9 (1.1–7.8)***	3.9 (2.4–6.2)	1.6 (0.7–3.5)
Q5	4.1 (2.7–6.2)	1.2 (0.6–2.3)	6.5 (3.1–13.5)	2.5 (1.0–6.6)	3.6 (2.1–6.2)	0.7 (0.3–1.7)
Q6	2.2 (1.5–3.3)	1.0 (0.6–1.8)	2.1 (0.9–4.6)	-	2.2 (1.4–3.4)	1.0 (0.5–2.0)
Q7	1.8 (1.3–2.5)	0.9 (0.5–1.4)	1.4 (0.7–2.7)	−	1.8 (1.2–2.7)	0.9 (0.5–1.5)
Q8	1.0 (0.7–1.4)	−	0.8 (0.4–1.5)	−	1.7 (1.1–2.7)	1.4 (0.6–1.9)
Q9	2.4 (1.5–3.6)	***2.5 (1.5–4.2)***	0.9 (0.3–2.3)	−	3.5 (2.1–5.7)	***3.2 (1.8–5.8)***
Q10	0.9 (0.6–1.4)	−	1.8 (0.8–4.0)	−	0.6 (0.4–1.1)	−
Q11	4.1 (1.6–10.6)	0.9 (0.3–3.3)	3.4 (0.7–15.5)	−	5.0 (1.4–18.1)	0.9 (0.2–5.0)
Q12	4.4 (3.1–6.3)	***2.8 (1.9–4.3)***	3.5 (1.6–7.6)	1.7 (0.6–4.8)	4.3 (2.8–6.6)	***3.2 (2.0–5.3)***
Q13	2.2 (1.2–4.0)	1.2 (0.5–3.0)	1.6 (0.6–4.0)	−	3.9 (1.5–10.2)	1.0 (0.3–4.2)
Q14	4.4 (1.7–11.3)	1.0 (0.3–3.7)	3.4 (0.7–15.5)	−	5.5 (1.2–19.7)	1.9 (0.2–15.8)
Q15	7.5 (4.0–13.8)	***3.0 (1.3–7.0)***	6.3 (2.1–19.1)	***4.9 (1.2–20.0)***	7.8 (3.7–16.5)	2.6 (0.9–7.9)
Q16	3.3 (2.2–5.1)	1.1 (0.6–2.0)	2.2 (1.0–4.8)	0.7 (0.2–2.0)	4.3 (2.5–7.6)	1.6 (0.8–3.4)
Q17	4.4 (1.9–10.0)	1.0 (0.3–3.0)	7.9 (1.5–40.5)	2.3 (0.3–17.8)	3.4 (1.3–9.0)	0.4 (0.1–1.8)
Q18	2.5 (1.7–3.6)	1.2 (0.8–2.0)	1.3 (0.6–2.9)	−	2.8 (1.8–4.4)	1.5 (0.8–2.6)
Q19	1.3 (0.9–1.8)	−	1.4 (0.7–2.7)	−	1.9 (1.2–2.9)	1.1 (0.6–1.9)
Q20	1.5 (1.0–2.0)	1.0 (0.6–1.6)	1.2 (0.6–2.4)	−	1.8 (1.2–2.6)	0.9 (0.5–1.5)
Q21	1.4 (1.0–2.0)	1.1 (0.7–1.7)	0.8 (0.4–1.5)	−	2.1 (1.4–3.2)	1.4 (0.7–2.5)
Q22	0.6 (0.4–0.9)	0.7 (0.5–1.0)	1.2 (0.6–2.5)	−	0.5 (0.4–0.8)	***0.6 (0.3–0.9)***
Q23	0.7 (0.5–1.1)	−	0.6 (0.3–1.4)	−	0.7 (0.5–1.2)	−
Age	1.0 (1.0–1.0)	1.0 (1.0–1.0)	0.8 (0.6–1.1)	0.7 (0.5–1.0)	1.0 (1.0–1.0)	1.0 (1.0–1.0)
Marital status	0.4 (0.3–0.6)	***0.4 (0.2–0.6)***	−	−	0.4 (0.3–0.7)	***0.4 (0.2–0.7)***

^a^ORs in bold italics are statistically significant.

## DISCUSSION

In this study, we aimed to identify the factors affecting the intention to participate in nuclear disaster activities among students and firefighters. This questionnaire survey revealed that students were significantly more willing to work in a nuclear disaster situation than firefighters. However, caution must be observed when interpreting these results. Students may not have been able to answer correctly because they lack experience related to actual disaster work. There is a possibility that they could not estimate the different outcomes of participating in nuclear disaster activities. In contrast, in this study, firefighters from a nuclear disaster-affected area had actual experiences related to nuclear disaster activities. We believe that the experiences mentioned above may have influenced the answers of firefighters. Compared with Hawaiian healthcare workers (40%) or Turkish students (42.4%), participants in our study had higher willingness to engage in a nuclear disaster activity (70.4% or 56.3%). The results of our study participants’ higher willingness percentage may be due to the actual experience of participation in the Fukushima nuclear disaster activities, so that our results cannot be compared with previous studies solely.

The students who were willing to work in a nuclear disaster situation wanted to work on nuclear disaster countermeasures and believed that firefighters should actively work in a nuclear disaster situation. According to the answers of “Q15: Firefighters should actively work in a nuclear disaster situation” (OR: 4.9), the students had a strong belief about the ideal image of a firefighter. Although a student’s decision might be strongly connected to social norms and they are more willing to be involved in nuclear disaster activities, the answers might not be applicable to those who have never experienced actual field activities. However, the following points can help increase human resources in a nuclear disaster situation. To maintain passion and motivation among students, they must receive continuous education and participate in public relations activities after graduation. In addition, they should actively obtain the basic knowledge and skills or prepare the practical manual for the countermeasure of nuclear disasters, to reflect the result of Q4 (OR: 2.9) in this study. Therefore, we should provide efficient opportunities to learn and prepare for a nuclear disaster.

We found four factors (marital status, self-confidence, incentive and family understanding) to be associated with the willingness to work in a nuclear disaster situation among firefighters. Their intentions to respond to a nuclear disaster situation might improve by facilitating activities that can build their self-confidence (Q9, OR: 3.2) and providing sufficient incentives (Q12, OR: 3.2). Training is a countermeasure that can increase self-confidence, and it is the only method with a validated efficacy. A previous study showed that firefighters receive insufficient training or exercises [6], thereby leading to insufficient knowledge of nuclear disasters and increased levels of anxiety related to radiation exposure. In another study on nurses, those who wanted to receive training were found to be significantly interested in nuclear disaster activities [7]. Thus, training about a radiological situation for disaster responders is essential and opportunities for training should be provided continuously. Moreover, regular nuclear disaster drills may increase the firefighters’ intention to participate in nuclear disaster activities, which may lead to an increase in human resources in this field.

In contrast, it is true that the intention to work in a nuclear disaster situation is inversely related to being married (OR: 0.4). In addition, although there was no significant effect of age, the intention tended to increase with age (OR: 1.0, 95% CI: 1.0–1.0) (Table 3). As age increases, not only does a firefighter’s self-confidence increase but also the likelihood of getting married—an unpreventable contradiction. To link self-confidence with participation in a nuclear disaster activity, we should provide firefighters with appropriate methods of communication with their spouse to ensure they understand and support their partner’s participation in disaster activities—a countermeasure for lack of understanding by family members.

Considering incentives, the provision of sufficient guaranty or insurance may be challenging because of legal issues. Regarding fire departments in Japan, the local government is the management body; therefore, the incentive depends on the management and economic situation of each local government. It will be necessary to consider establishing national standards and support in the future and take other associated measures.

Moreover, intention to participate is correlated to the fact that it is difficult to increase understanding among family members about the nature of the work (Q22, OR: 0.6). Thus, if families have sufficient understanding, these professionals could willingly engage in nuclear disaster activities. This result implies that educating or providing accurate information about radiation to not only firefighters but also their families and the general public is extremely essential.

Finally, to increase the firefighters’ intention to participate in nuclear disaster activities, regular training opportunities must be provided and individuals must be taught how to provide adequate explanation to their family members during a training course. Moreover, local governments, fire departments or other organizations must provide substantial incentives in the future. It is difficult to estimate the effectiveness of these countermeasures. We also perceive this as our study limitation. A further study confirming the effectiveness of these countermeasures is expected in the future.

## References

[1] Lanzilotti SS, Galanis D, Leoni N et al. Hawaii medical professionals assessment. Hawaii Med J 2002;61:162–73. 12242780

[2] Qureshi K, Gershon RR, Sherman MF et al. Health care workers’ ability and willingness to report to duty during catastrophic disasters. J Urban Health 2005;82:378–88. 16000654 10.1093/jurban/jti086 PMC3456052

[3] Kaya E, Altintas H. Willingness of firefighting program students to work in disasters-Turkey. Prehosp Disaster Med 2018;33:13–22. 29223186 10.1017/S1049023X17007087

[4] Odai ED, Azodo CC, Chhabra KG. Disaster management: Knowledge, attitude, behavior, willingness, and preparedness among Nigerian dentists. Prehosp Disaster Med 2019;34:132–6. 30968803 10.1017/S1049023X19000074

[5] Smith EC, Burkle FM Jr, Archer FL. Fear, familiarity, and the perception of risk: A quantitative analysis of disaster-specific concerns of paramedics. Disaster Med Public Health Prep 2011;5:46–53. 21402826 10.1001/dmp.10-v4n2-hre10008

[6] Rebmann T, Charney RL, Loux TM et al. Firefighters' and emergency medical service personnel’s knowledge and training on radiation exposures and safety: Results from a survey. Health Sec 2019;17:393–402. 10.1089/hs.2019.0086 PMC6791471 31593509

[7] Yamada Y, Orita M, Shinkawa T et al. Nurses’ interest in nuclear disaster medicine: Future capacity building. J Radiat Res 2019;60:333–4. 30968931 10.1093/jrr/rrz008 PMC6530613

